# Identification of Progression‐Associated Biomarkers in Lung Cancer Based on the Integrated Analysis of RNA Sequencing Data From Platelets and Tumor Tissues

**DOI:** 10.1155/ijog/3979354

**Published:** 2025-08-28

**Authors:** Liancheng Lin, Xuemei Wu, Kangmei Dong, Maoli Chen, Jianxiong Xu, Daxiong Han, Yanyan Yang, Xue Yi, Chih-Jung Chang, Guodong Ye, Mingyao Ke

**Affiliations:** ^1^ Department of Respiratory Centre, The Second Affiliated Hospital of Xiamen Medical College, Xiamen, Fujian, China; ^2^ Xiamen Lifeint Technology Co. Ltd., Xiamen, Fujian, China; ^3^ School of Chemistry and Chemical Engineering, Changji University, Changji, Xinjiang, China, yau.edu.cn; ^4^ Third Institute of Oceanography, Ministry of Nature Resources, Xiamen, Fujian, China; ^5^ School of Medical Sciences Xiamen Medical College, Institute of Respiratory Diseases Xiamen Medical College, Xiamen, Fujian, China; ^6^ Medical Research Center and Xiamen Chang Gung Allergology Consortium, Xiamen Chang Gung Hospital, Xiamen, Fujian, China, cgmh.com.cn; ^7^ School of Medicine, Huaqiao University, Quanzhou, Fujian, China, hqu.edu.cn; ^8^ Fujian Collaborative Innovation Center for Precision Medicine in Respiratory Diseases, Xiamen, Fujian, China

**Keywords:** biomarkers, lung cancer, platelet, prognosis, tumor-educated platelets

## Abstract

**Objective:** This study is aimed at exploring disease progression–associated genes from platelet‐derived genes and at investigating their underlying roles in prognostic outcomes in lung cancer.

**Methods:** Platelet RNA sequencing (RNA‐seq) from healthy controls (*n* = 81) and lung cancer patients at early (*n* = 102) and advanced stages (*n* = 65) was conducted, and the genes from which that continuously changed with disease progression were screened by differential analysis and WGCNA. RNA‐seq and survival data of LUAD cohort from TCGA database was utilized for prognostic investigation. GSE31210 and GSE18842 datasets from GEO database were utilized for validation of gene expression and prognosis. The immunedeconv package and ESTIMATE algorithm were employed for investigation of immune status. Gene mutation was evaluated based on the cBioPortal database. Drug sensitivity was assessed based on the GDSC database.

**Results:** Totally, 53 platelet‐derived genes that were persistently dysregulated along with the progression from normal to early and then advanced were identified. These 53 genes were primarily enriched in ribosome biogenesis–related functions. Five prognostic genes, including HPSE, DENND1C, GRWD1, HLA‐DQA1, and PDXK, were identified to further develop a risk signature, which exhibited moderate power for forecasting the prognosis of lung cancer patients in training, testing, and validation sets. In addition, a high‐risk signature score was linked to low infiltrating levels of most immune cells and a high tumor purity in the tumor microenvironment, as well as low IC50 values to several common chemotherapeutics, such as docetaxel, gefitinib, and erlotinib. Moreover, energy metabolism and proliferation‐related pathways were activated, while immune‐related pathways were inactivated in the high‐risk group. Among the five prognostic genes, HLA‐DQA1 harbored a relatively higher alteration frequency in LUAD (3%, alteration type: amplification).

**Conclusion:** The five platelet‐derived prognostic genes might be potential targets or biomarkers in lung cancer.

## 1. Introduction

Lung cancer is the foremost cause of cancer‐related incidence and mortality [1, 2]. For early‐stage lung cancer, surgical resection is the commonly recommended treatment. However, even after a complete resection, around 50% of patients with Stage I–IIIA NSCLC will relapse and die within 5 years [3]. As public awareness has grown and lung cancer screening has been more widely implemented, the rate of early‐stage lung cancer has risen sharply [4]. Despite this, more than 70% of patients are diagnosed once their tumors have progressed to advanced stages, and many of them are not suitable for surgical treatment [5]. The 5‐year survival rate for lung cancer patients diagnosed at an early stage is approximately 70% or higher, but this rate falls to below 10% for those diagnosed at an advanced stage [6]. Therefore, the most promising approach to improving lung cancer prognosis is to identify early‐stage lung cancer patients who are prone to progression, which facilitates optimizing personalized therapeutic strategies.

Blood platelets, the second most abundant cells in peripheral blood originating from megakaryocytes, have been proposed as major modulators in tumorigenesis and progression [7]. Increased counts of circulating platelets are dramatically linked to the onset and prognosis of a variety of malignancies [8], especially lung cancer, because a considerable fraction of platelets is generated in the lung [9]. Accumulating evidence has revealed that platelets can dynamically interact with tumor cells and continually enrich and absorb tumor‐specific substances during circulation, resulting in both indirect or direct changes to their transcriptome [10]. Such unique transcriptomic signatures from platelets allow for selecting biomarkers for cancer early detection and prognosis prediction in tumors [11]. Therefore, the platelet transcriptome has been considered a valuable source of cancer biomarkers [12, 13], and numerous studies have constructed platelet‐related prognostic signatures to forecast the prognosis of multiple cancers [14–17], including lung cancer [18]. However, the signatures in the public studies for lung cancer are often obtained from only a single source of sample type, which might lead to insufficient specificity and sensitivity.

To address this issue, we conducted platelet RNA sequencing (RNA‐seq) from healthy controls and lung cancer patients at early and advanced stages to excavate the genes that continuously changed with disease progression. Besides, the platelet RNA‐seq was analyzed by integrating with the RNA‐seq from tumor tissues to screen potential biomarkers for lung cancer. Overall, this study was aimed at exploring key platelet genes related to tumor progression and developing a platelet‐derived gene signature for predicting the prognosis of patients with lung cancer, which could aid in patient stratification and hold promise for personalized treatment approaches.

## 2. Methods

### 2.1. Blood Sample Collection

Blood samples from 248 participators were collected for platelet RNA‐seq, including 102 patients with early and 65 patients with advanced lung cancer in The Second Affiliated Hospital of Xiamen Medical College, as well as 81 healthy blood donors (Table 1). Informed consent was obtained, and the ethics committee of The Second Affiliated Hospital of Xiamen Medical College permitted the application of the human specimens in this research (Approval No. 2018013).

**Table 1:** The demographic data of the clinical samples.

	**Advanced (** **n** = 65**)**	**Early (** **n** = 102**)**	**Normal (** **n** = 81**)**	**p** **value**
Gender, *n* (%)				0.176
Male	42 (64.6)	51 (50.0)	46 (56.8)	
Female	23 (35.4)	51 (50.0)	35 (43.2)	
Age, mean (SD)	61.14 (9.60)	61.83 (10.17)	59.75 (13.26)	0.452

Abbreviation: SD, standard deviation.

### 2.2. Blood Platelet Isolation

For RNA‐seq, platelets were first collected by means of a magnetic separating method [19]. Specifically, to obtain the platelet‐rich plasma (PRP), the whole blood sample was centrifuged at 200×*g* for 20 min. The obtained PRP was then suspended in EDTA, and platelets were further collected from PRP after centrifuging at 1000×*g* for 10 min. Afterwards, the collected platelet pellets were resuspended in PBS, 0.5% bovine serum albumin, 2.5 mM of EDTA, and 175 ng·mL^−1^ of prostaglandin E1 (to minimize ex vivo platelet activation). Human CD45 and CD235a MicroBeads reagents were added to the platelet suspension (mainly to remove residual leukocytes and red blood cells, respectively) for 15 min of incubation; magnetic separating was conducted to collect platelets.

### 2.3. Blood Platelet RNA Isolation and Sequencing

Total RNAs were extracted from the above purified platelets utilizing the Trizol method, followed by determination of RNA concentration and purity on a spectrophotometer. The platelet RNAs were amplified to generate the complementary DNA (cDNA), which was then utilized for library construction. Following purification, the library was sequenced on the NovaSeq 6000 (Illumina Inc., San Diego, California, United States) platform, with the PE150, 6G Raw Base sequencing strategy.

### 2.4. Publicly Available Datasets

The RNA‐seq and clinical data for lung adenocarcinoma in TCGA database (TCGA‐LUAD) were acquired from the UCSC website. The TCGA‐LUAD cohort contained 583 samples (524 LUAD and 59 normal), which were utilized for prognostic analysis and other risk score–related analyses. Besides, two microarray datasets, GSE31210 and GSE18842, were obtained from the GEO database. Both of these datasets were employed for verification, with GSE31210 for prognosis and GSE18842 for gene expression. The detailed information for the used datasets is summarized in Table 2.

**Table 2:** The detailed information for the used datasets in this study.

**Datasets**	**Platforms**	**Data type**	**Sample type**	**Sample size**	**Usage**
Platelet RNA‐seq	GRCh38	mRNA	Platelet	248 (81 normal/102 early/65 advanced)	Train
TCGA‐LUAD	GRCh38	mRNA	Lung tissue	583 (524 LUAD and 59 normal)	Prognostic
GSE31210	GPL570	mRNA	Lung tissue	246 (226 LUAD and 20 normal)	Prognostic verification
GSE18842	GPL570	mRNA	Lung tissue	91 (46 LUAD and 45 normal)	Expression verification

Abbreviation: LUAD, lung adenocarcinoma.

### 2.5. Screening of Differentially Expressed Genes (DEGs)

The DESeq2 package (Version 1.42.1) was employed to conduct differential analysis in early versus normal, advanced versus normal, and advanced versus early, followed by Benjamini & Hochberg corrections for multiple tests. Subsequently, DEGs were selected with the threshold of *p*.adj < 0.05 and |log_2_FC| > 0.5.

### 2.6. Weighted Gene Coexpression Network Analysis (WGCNA)

The WGCNA package (Version 1.72‐5) was run to identify disease progression–associated gene modules from platelet RNA‐seq data. Specifically, to establish a scale‐free network, a soft threshold power was first determined by calculating the square value of the correlation coefficient and average connectivity under each power. Afterwards, gene hierarchical clustering and dynamic tree cutting (minmoduleSize = 100) were conducted to identify highly correlated gene modules. Next, the relationships of each module eigengene with clinical phenotypes (normal, early, and advanced) were calculated to identify disease progression–associated module genes (correlation coefficient gradually increased or decreased with disease progression).

### 2.7. Screening of Disease Progression–Associated DEGs

The overlapped genes between DEGs and the disease progression–associated module genes in WGCNA were screened by the ggvenn package (Version 0.1.10), which were considered as the disease progression–associated DEGs. The clusterProfiler package (Version 4.4.4) was employed to conduct enrichment analysis to discover the gene ontology (GO) terms and KEGG pathways for these overlapped genes.

### 2.8. Establishment and Assessment of Risk Prognostic Model

The TCGA‐LUAD cohort was utilized for prognosis analysis. Briefly, with the aid of the caret package (Version 6.0‐94), the patients in this cohort were first assigned into training and test sets with an assignment ratio of 6:4. Besides, GSE31210 was selected as an external validation set. In the training set, the univariate Cox survival analysis was conducted for the above screened overlapped genes to discover their prognosis associations. Subsequently, LASSO regression was conducted to further shrink the number of prognostic genes based on the minimum criteria (lambda.min), which was conducted for 1000 cycles with tenfold cross‐validation, followed by screening with the multivariable Cox step regression. Afterwards, the risk model was established as per the following equation:

Risk score=∑n=1ncoefi∗Xi.

In the equation, coef and *X* refer to the coefficient and the expression value of each gene. After calculating the risk score for each patient in three sets, we categorized these patients into high and low‐risk groups as per the optimal threshold (cut‐off value) determined by the survminer package (Version 0.4.9). KM survival curves were plotted with the survminer package, and the performance of the model for predicting 1–5 years’ survival was assessed by plotting ROC curves with the timeROC package (Version 0.4).

### 2.9. Assessment for Immune Status

We assessed the infiltrating fractions of diverse cell types in samples utilizing the immunedeconv package (Version 2.1.0) [20], which contained EPIC, ConsensusTME, quanTIseq, TIMER, ABIS, and xCell, these six algorithms. Correlations across risk score with the level of each cell type were then revealed. Moreover, the ESTIMATE algorithm was run to infer the stromal and immune scores in tumor tissues, which could also indirectly reflect the tumor purity.

### 2.10. Gene Set Enrichment Analysis (GSEA)

To discover the dysregulated pathways along with the changes of risk score, we conducted GSEA [21, 22]. Specifically, we first sorted genes according to the fold changes in differential analysis in high‐ versus low‐risk groups, and then, GSEA was run with the KEGG gene set as enrichment references. Those with *p*.adj < 0.05 and |normalized enrichment score (NES)| > 1 were considered as significant pathways.

### 2.11. Gene Mutations

The cBioPortal database (https://www.cbioportal.org/) was employed to explore the mutated status of prognostic genes in the model. Moreover, the GSCALite (https://guolab.wchscu.cn/GSCA/#/) database was employed to further explore the relationship of prognostic genes with the oncogenic pathways.

### 2.12. Drug Sensitivity

To explore the linkage of risk score with drug sensitivity, we first quantified the IC50 value of each LUAD patient to common chemotherapeutics using oncoPredict package (Version 0.2). Next, correlation analysis was carried out to calculate the correlations of IC50 values with the risk score and the expression of each prognostic gene.

### 2.13. Statistical Analysis

All analyses were conducted using R language (Version 4.2.3). Overall survival between groups was compared by log‐rank test. Correlation analysis was assessed by Spearman’s correlation coefficient. Comparison in terms of key gene expression, four kinds of scores calculated by ESTIMATE algorithm, and IC50 value of drugs between two groups was conducted using the Wilcoxon rank sum test.  ^∗^ *p* < 0.05,  ^∗∗^ *p* < 0.01,  ^∗∗∗^ *p* < 0.001, and  ^∗∗∗∗^ *p* < 0.0001.

## 3. Results

### 3.1. Screening of Genes That Dysregulated With Lung Cancer Progression

Based on platelet RNA‐seq data, we performed differential analysis among normal, early, and advanced in these three groups. Compared to normal samples, there were 1721 DEGs, including 732 up‐ and 989 downregulated genes in early lung cancer (Figure 1a). Besides, there were 2572 DEGs (674 up‐ and 1898 downregulated) in advanced versus normal (Figure 1b) and 4116 DEGs (1145 up‐ and 2971 downregulated) in advanced versus early groups (Figure 1c). Heatmaps of the top 50 DEGs indicated that expression of these DEGs could clearly distinguish samples between different groups (Figures 1d, 1e, and 1f). The screened DEGs were considered to be the genes that were dysregulated along with the disease progression.

Figure 1Differential expression analysis. Volcano plots showing the genes that are differentially expressed in (a) early versus normal, (b) advanced versus normal, and (c) advanced versus early. The gray dots represent the genes with no differential expression between groups. Blue and red dots represent the downregulated and upregulated genes, respectively. Heatmaps showing the expression pattern of the top 50 differential genes in (d) early versus normal, (e) advanced versus normal, and (f) advanced versus early. Blue and red in the lower block of the heatmap indicate a relatively lower or higher expression of genes than the control group.

**Figure:**
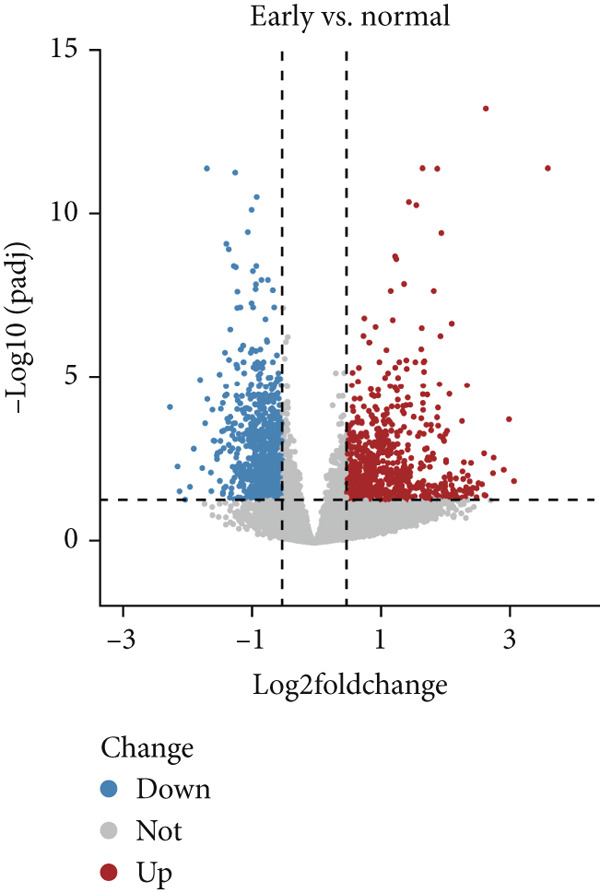
(a)

**Figure:**
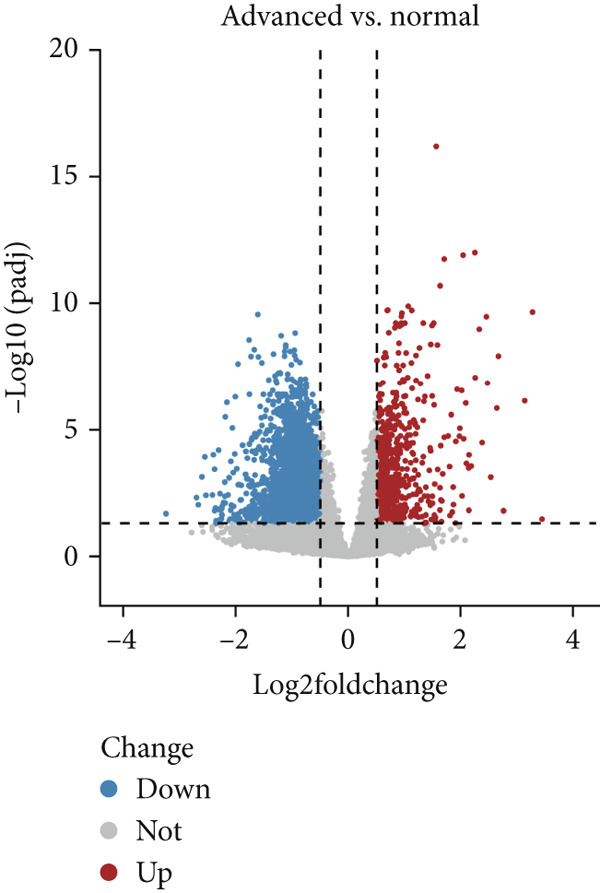
(b)

**Figure:**
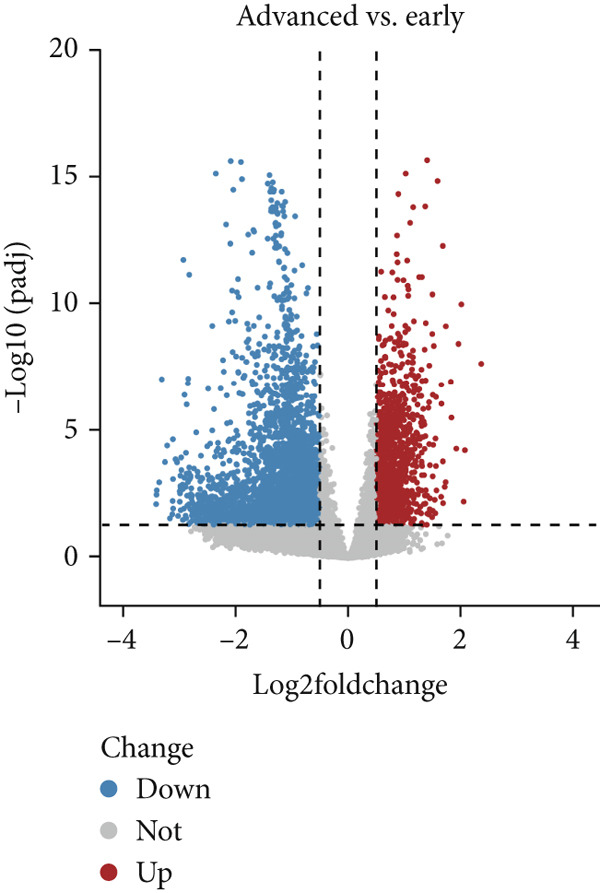
(c)

**Figure:**
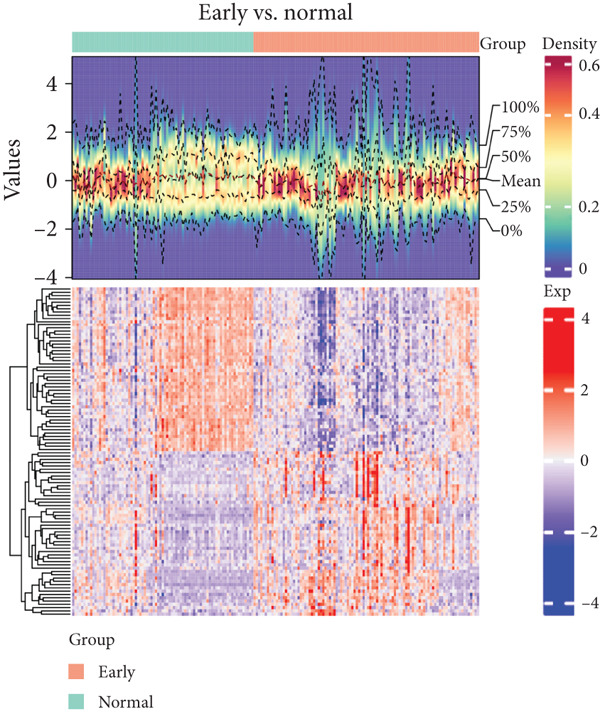
(d)

**Figure:**
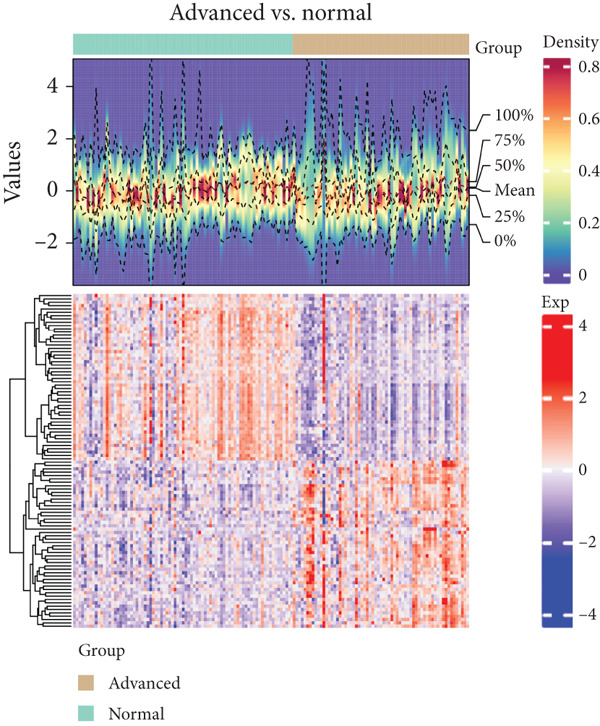
(e)

**Figure:**
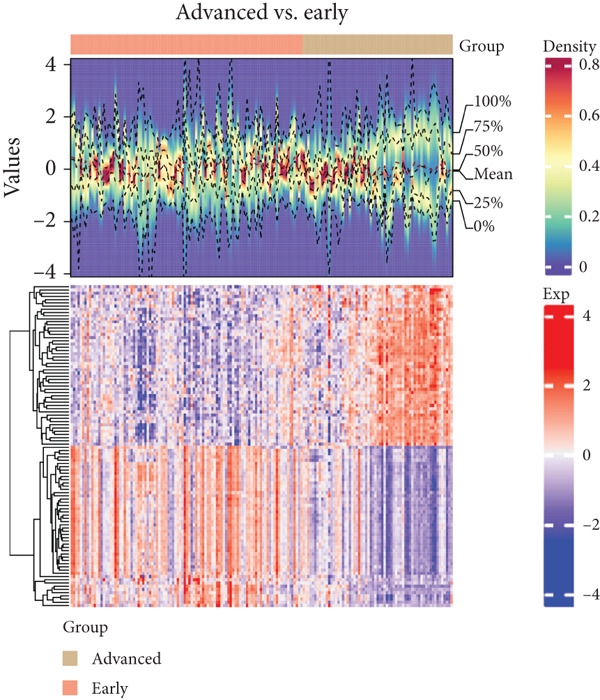
(f)

### 3.2. Lung Cancer Progression–Associated Gene Modules in WGCNA

**Figure 2:**
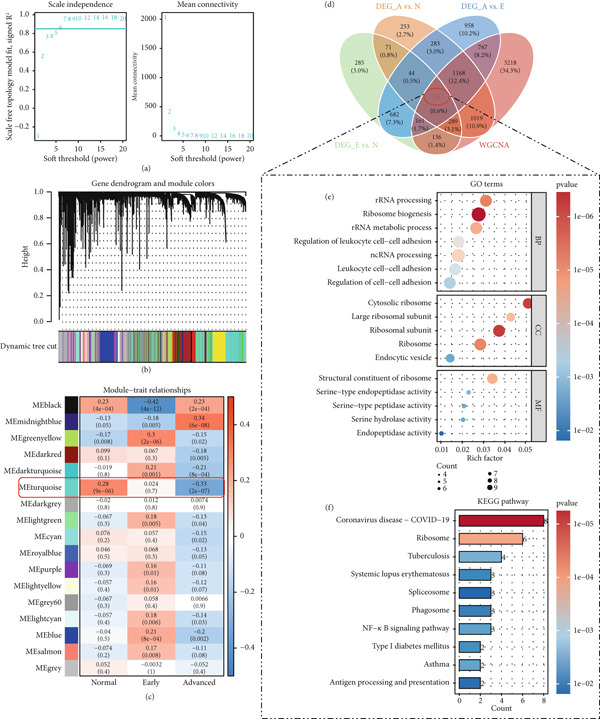
Disease progression–associated differential genes. (a) The scale‐free topology model fit (left) and mean connectivity (right) for determination of soft threshold in WGCNA. (b) The cluster dendrogram showing the gene modules in WGCNA. Each color represents a gene module. (c) Module–trait correlations. The numbers in each box represent the correlation coefficient (upper) and the specific *p* value (lower). (d) Venn analysis showing the overlapped genes among three sets of DEGs and WGCNA module genes. (e) The significantly enriched gene ontology terms for the 53 overlapped genes screened by Venn analysis; the size of the circle represents the gene number in corresponding functional terms, and the color of the circle represents the significance (*p* value) of corresponding functional terms. (f) The significantly enriched KEGG pathways for the 53 overlapped genes screened by Venn analysis. The length of the block represents the gene number in corresponding pathway, and the color of the block represents the significance (*p* value) of corresponding pathway.

WGCNA was conducted based on platelet RNA‐seq data. Firstly, sample clustering revealed three outlier samples, which were removed from the WGCNA analysis. Based on the scale‐free topological fit index *R* ^2^ reaching 0.85 for the first time, a soft threshold power of 6 was selected to approach the scale‐free network distribution (Figure 2a). Further clustering analysis identified 17 gene modules (Figure 2b), and the “turquoise” module exhibited correlations with normal (*r* = 0.28), early (*r* = 0.024), and advanced (*r* = −0.033) traits, and the correlations gradually decreased along with early and advanced diseases (Figure 2c). There were 6811 genes in the “turquoise” module, which were disease progression–associated genes.

The intersection of the three sets of DEGs and the 6811 genes in WGCNA was screened by Venn analysis, which generated 53 overlapped genes (Figure 2d), that is, lung cancer progression–associated DEGs. These genes were primarily involved in ribosome‐related GO terms, such as rRNA processing, rRNA metabolic process, and ribosome biogenesis, as well as in leukocyte cell–cell adhesion (Figure 2e). Similarly, ribosome and antigen processing and presentation pathways were also markedly enriched for these genes (Figure 2f).

### 3.3. Risk Prognostic Model Based on Disease Progression–Associated Genes

To explore the prognostic associations of disease progression–associated genes, the TCGA‐LUAD cohort was utilized. Firstly, with the cut‐off value of *p* < 0.05 and the proportional hazards test over 0.5 (no violations of the assumptions of Cox proportional‐hazard regression), seven genes were identified to be associated with prognosis in univariate survival analysis (Table 3), including EI24, HPSE, DENND1C, GRWD1, ICAM3, HLA‐DQA1, and PDXK. Among these seven genes, no one was shrunken by LASSO regression (Figure 3a). Further multivariate Cox step regression determined five genes, including HPSE, DENND1C, GRWD1, HLA‐DQA1, and PDXK (Figure 3b). Their expression trends were confirmed in different datasets. As expected, in the TCGA‐LUAD cohort, the expression of GRWD1 and HPSE was enhanced in tumors while the expression of PDXK, DENND1C, and HLA‐DQA1 was reduced (Figure 3c). In the GSE18842 external dataset, a consistent expression trend between groups was observed for DENND1C, GRWD1, HPSE, and HLA‐DQA1, but no significance was found for HLA‐DQA1 (Figure 3d). In platelet RNA‐seq data, the expression of DENND1C, GRWD1, and PDXK was gradually reduced from normal tissue to early and even advanced tumors, while the expression of HPSE was gradually elevated (Figure 3e).

**Table 3:** Results of univariate Cox survival analysis.

**Gene**	**HR**	**HR.95L**	**HR.95H**	**p** **value**	**PH** **p** **value**
HPSE	1.39	1.09	1.79	0.009	0.48
HLA‐DQA1	0.88	0.78	0.99	0.038	0.346
EI24	1.74	1.16	2.61	0.008	0.096
DENND1C	0.66	0.48	0.91	0.011	0.726
ICAM3	0.74	0.57	0.96	0.025	0.353
GRWD1	1.69	1.08	2.64	0.022	0.21
PDXK	1.34	1.01	1.78	0.043	0.297

Figure 3Screening of key prognostic genes. (a) The coefficient distribution (left) and partial likelihood deviance (right) of LASSO regression. (b) Results of the multivariate Cox step regression. Expression of prognostic genes between tumor and normal samples in (c) TCGA and (d) GSE18842 dataset. (e) Expression of prognostic genes in normal, early, and advanced samples based on platelet RNA‐seq data.

**Figure:**
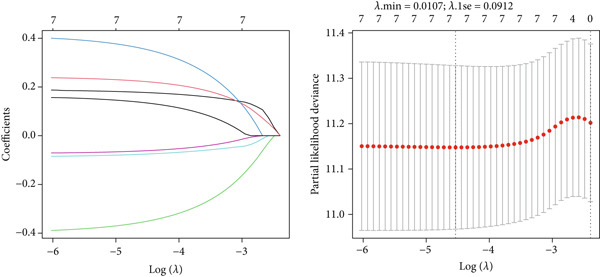
(a)

**Figure:**
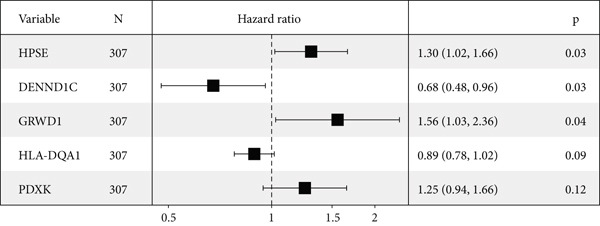
(b)

**Figure:**
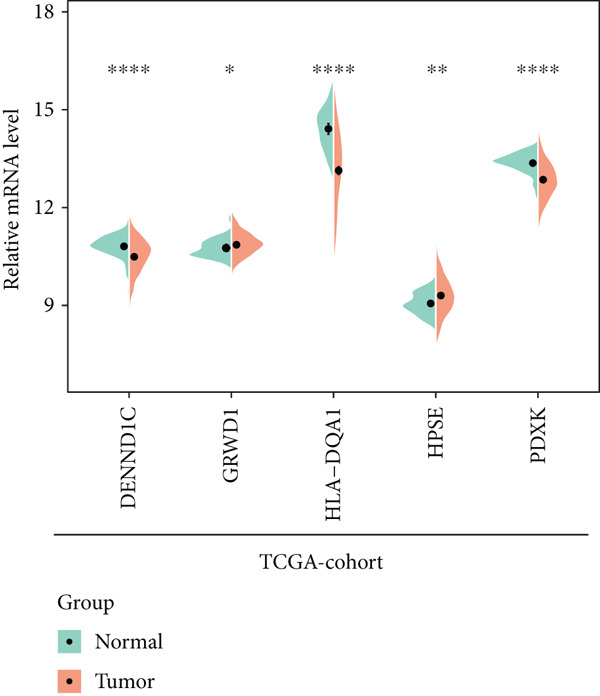
(c)

**Figure:**
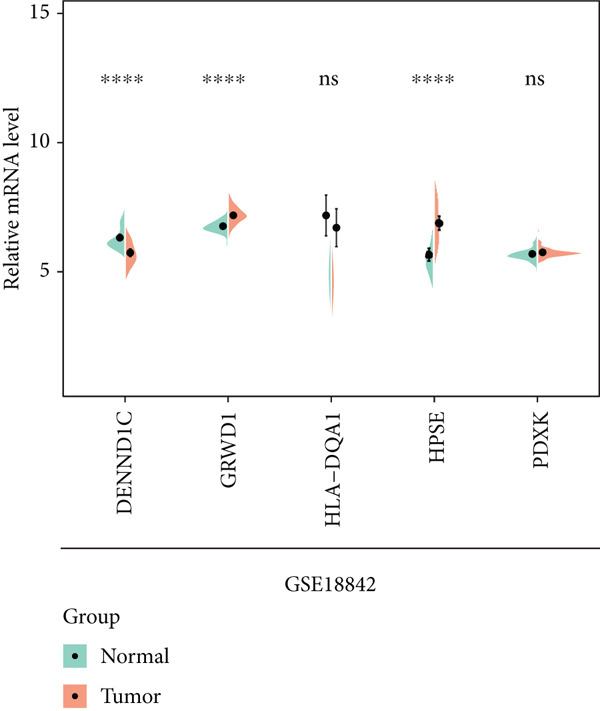
(d)

**Figure:**
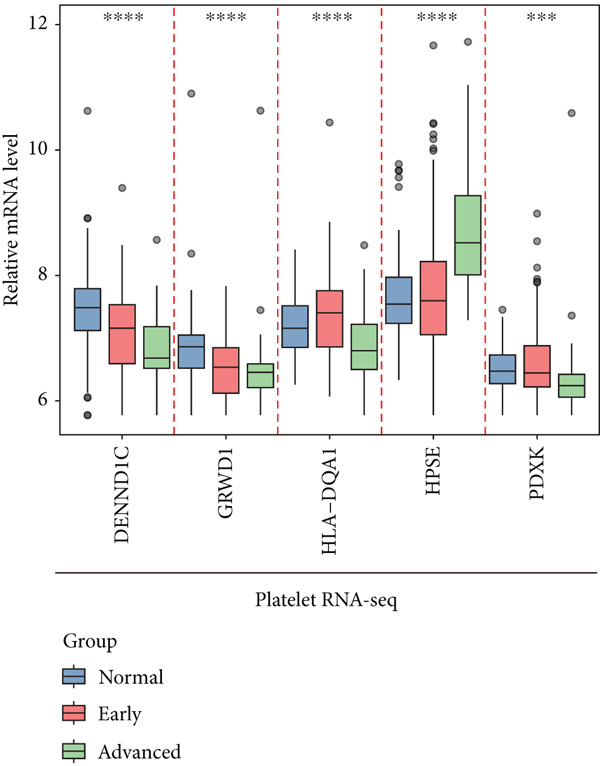
(e)

**Figure 4:**
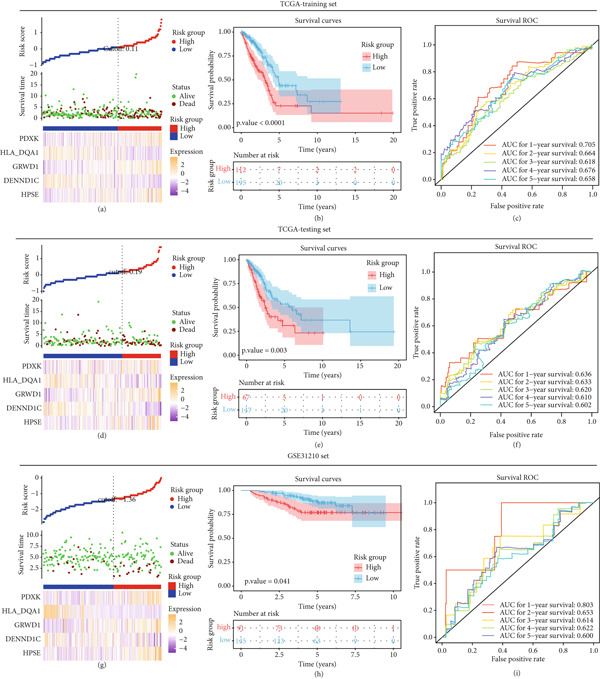
Establishment and assessment of risk prognostic model. Establishment and assessment of risk model in (a–c) TCGA‐training, (d–f) TCGA‐testing, and (g–i) GSE31210 sets. (a, d, g) The scatterplot of risk score based on risk groups (upper) and survival time (middle) and the expression heatmaps (bottom) of prognostic genes in risk groups. Each dot in the scatterplot indicates each sample in the dataset. The cut‐off value in the scatterplot of risk score based on risk groups was the optimal threshold calculated by the survminer package; (b, e, h) the survival curves showing the survival probability between two risk groups; (c, f, i) the ROC curves for assessing the power of the risk model for predicting 1–5 years’ survival.

These five genes were utilized for risk model establishment to evaluate the risk score of each patient. Risk score was calculated as follows: risk score = (0.22243) × expression of HPSE + (− 0.35265) × expression of DENND1C + (0.35536) × expression of GRWD1 + (− 0.06174) × expression of HLA − DQA1 + (0.13531) × expression of PDXK. In the TCGA‐training set, the risk model could assign patients with exact risk scores, thereby dividing those into high‐ and low‐risk groups based on the optimal cut‐off value of 0.11 calculated by the survminer package. The expression of PDXK, GRWD1, and HPSE gradually rose, while the other two gradually reduced along with the increase in risk score (Figure 4a). KM curves indicated that the divided high‐risk patients were more likely to have an unsatisfactory survival probability than those with low risk (Figure 4b). Besides, the AUC of this model for predicting 1–5 years’ survival was over 0.6, implying a moderate predictive power (Figure 4c). Similar observations occurred in both the TCGA‐testing set (Figures 4d, 4e, and 4f) and in the GSE31210 external set (Figures 4g, 4h, and 4i). Correlations with clinical variates (Table 4) displayed that there was a higher proportion of patients with advanced tumors in high‐risk groups (high vs. low of 45.5% vs. 61.5 for Stage I and 25% vs. 13.9% for Stage III–IV) and lymphatic metastasis (high vs. low of 28.6% vs. 14.9% for N1), whereas a lower proportion of patients had small or noninvasive tumors (high vs. low of 20.5% vs. 39.0% for T1).

**Table 4:** Correlations of risk score with clinical features.

	**High risk (** **n** = 112**)**	**Low risk (** **n** = 195**)**	**p** **value**
Gender			
Female	51 (45.5%)	112 (57.4%)	0.058
Male	61 (54.5%)	83 (42.6%)	
Age			
Mean (SD)	62.7 (10.6)	66.5 (9.74)	**0.002**
Median [min, max]	63.0 [33.0, 87.0]	67.0 [38.0, 88.0]	
Missing	3 (2.7%)	3 (1.5%)	
Stage			
I	51 (45.5%)	120 (61.5%)	**0.048**
II	32 (28.6%)	45 (23.1%)	
III	20 (17.9%)	21 (10.8%)	
IV	8 (7.1%)	6 (3.1%)	
Unknown	1 (0.9%)	3 (1.5%)	
Pathologic_T			
T1	23 (20.5%)	76 (39.0%)	**0.015**
T2	76 (67.9%)	95 (48.7%)	
T3	9 (8.0%)	17 (8.7%)	
T4	3 (2.7%)	6 (3.1%)	
Unknown	1 (0.9%)	1 (0.5%)	
Pathologic_N			
N0	60 (53.6%)	139 (71.3%)	**0.004**
N1	32 (28.6%)	29 (14.9%)	
Unknown	20 (17.9%)	27 (13.8%)	
Pathologic_M			
M0	78 (69.6%)	130 (66.7%)	0.139
M1	8 (7.1%)	6 (3.1%)	
Unknown	26 (23.2%)	59 (30.3%)	

*Note:* The *p* values marked in bold represent statistically significant differences between the groups.

### 3.4. Risk Score Correlated With Tumor Immune Status and Therapy Sensitivity

By means of the six algorithms in the immunedeconv package, we inferred the infiltrating fractions of diverse immune cells in tumor tissues to further assess their correlations with risk score (Figure 5a). Specifically, risk score was observed to negatively correlate (correlation coefficient < 0) with majority of immune cells, including most effector T lymphocytes, B cells, macrophages, and neutrophils. Positive correlations across risk score with the infiltration levels were observed only in several cell types, such as common lymphoid progenitor and Th1/2 CD4+ T cells in the xCell algorithm, as well as the memory CD8+ T cells and gamma delta T cells in the ABIS algorithm (Figure 5a). These results implied that those with a high‐risk score were more likely to have an immunosuppressive tumor microenvironment (TME). This was also confirmed by the results of the ESTIMATE analysis, which proposed that there was a high tumor purity and a low immune/stroma score in the TME of high‐risk patients (Figure 5b). Sensitivity of patients to common chemotherapeutics was further estimated. Positive correlations were observed from risk score to IC50 of most drugs, indicating risk scores tended to link with a high drug sensitivity (Figure 6a). Among the five prognostic genes, the expression of four genes correlated with IC50 to several drugs, particularly HLA‐DQA1, which exhibited negative correlations with the IC50 to multiple drugs (Figure 6a). We further displayed the top 10 drugs whose IC50 markedly differed between the two risk groups. High‐risk patients had low IC50 to docetaxel, gefitinib, erlotinib, and lapatinib, indicating they might benefit from these drugs (Figure 6b).

Figure 5Correlations of risk score with immune status. (a) Correlation results between risk score and the infiltration levels of various immune cells. Different colors in the axis of ordinates represent the immune cells of different algorithms. The dots represent the exact correlation coefficient; (b) the boxplots showing the immune, stromal scores, and tumor purity between two risk groups.

**Figure:**
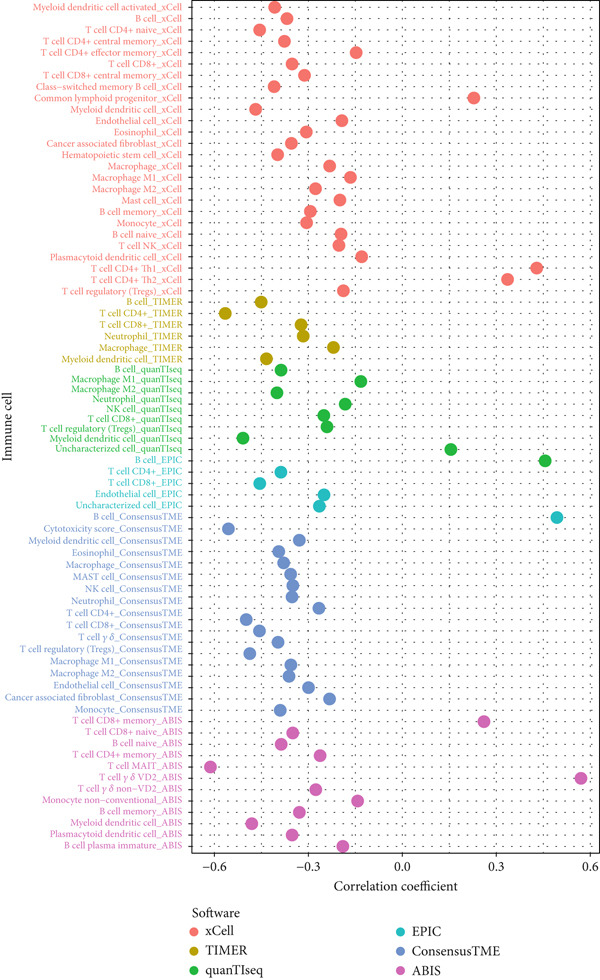
(a)

**Figure:**
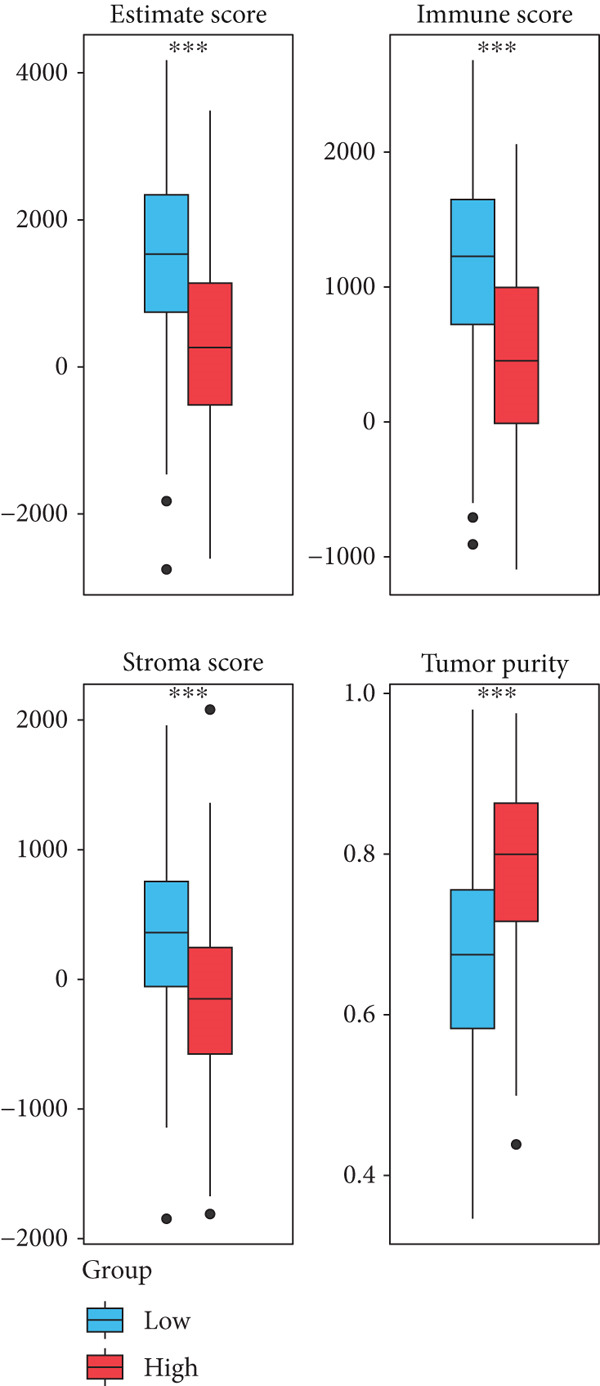
(b)

Figure 6Differences on drug sensitivity and pathways between risk groups. (a) Correlation results between drug sensitivity (shown by IC50 values) and risk score as well as prognostic genes. The numbers in each box represent the correlation coefficient, and “∗” represents *p* value; (b) the top drugs that showed significant differences in their IC50 values between risk groups; (c) the top five activated (marked in purple) or inactivated pathways (marked in orange) between risk groups in GSEA.

**Figure:**
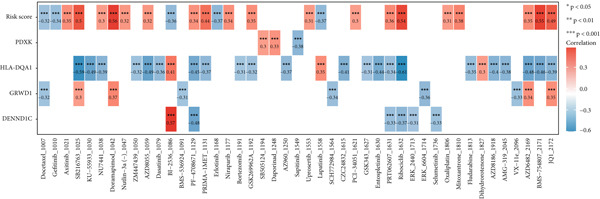
(a)

**Figure:**
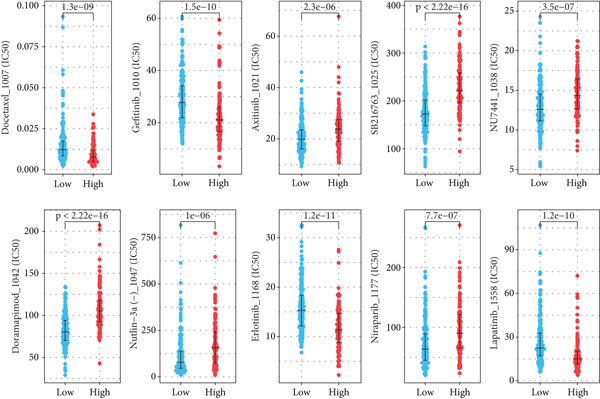
(b)

**Figure:**
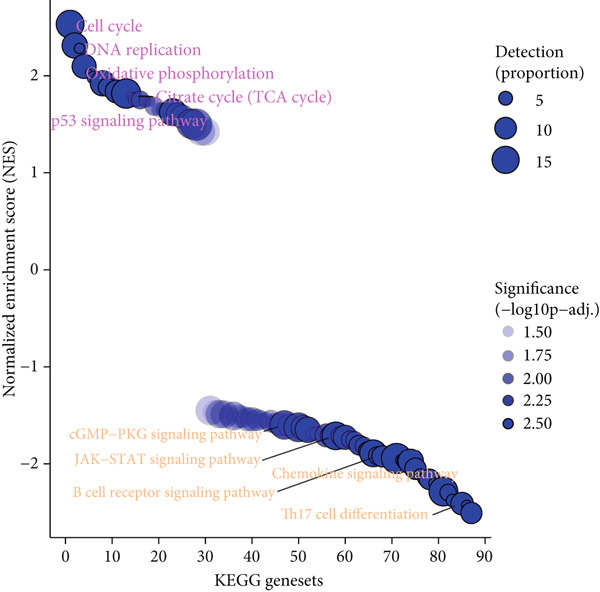
(c)

### 3.5. Pathways That Dysregulated Along With Risk Scores

KEGG pathways that dysregulated along with the risk scores were further explored, and 87 pathways were revealed. The top five up‐ and downregulated pathways are displayed in Figure 6c. Specifically, energy metabolism and proliferation‐related pathways, cell cycle, oxidative phosphorylation, DNA replication and citrate cycle (TCA cycle), and p53 signaling pathway were activated in the high‐risk group, whereas immune‐related pathways, B cell receptor signaling pathway, chemokine signaling pathway, Th17 cell differentiation, cGMP−PKG signaling pathway, and JAK–STAT signaling pathway were inactivated in the high‐risk group.

### 3.6. Mutation Profile and Potential Function of Prognostic Genes in LUAD Patients

Frequency and type of genetic alteration for the five prognostic genes in the context of LUAD were further assessed (Figure 7a,b). HLA‐DQA1 harbored a relatively higher alteration frequency in LUAD, reaching 3%, and the alteration type of HLA‐DQA1 was limited to amplification. The amplification and missense mutation in PDXK and GRWD1 caused a total of 1% and 0.8% genetic alteration, respectively. The relationship of prognostic genes with the common oncogenic pathways was also explored (Figure 7c). Expression of GRWD1 was linked to the activation of apoptosis, cell cycle, and EMT pathways, while the expression of both HLA‐DQA1 and DENND1C was linked to inhibition of the cell cycle pathway. Besides, the expression of HLA‐DQA1 was also linked to the activation of EMT as well as hormone androgen and estrogen receptor.

Figure 7Genetic variations of prognostic genes. (a, b) The frequency and types of genetic variations in prognostic genes in tumor samples; (c) correlations of prognostic genes and the activated (marked in red) or inhibited status (marked in blue) of oncogenic pathways.

**Figure:**
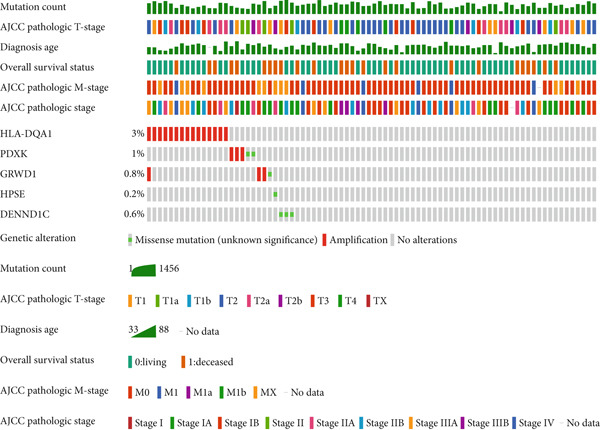
(a)

**Figure:**
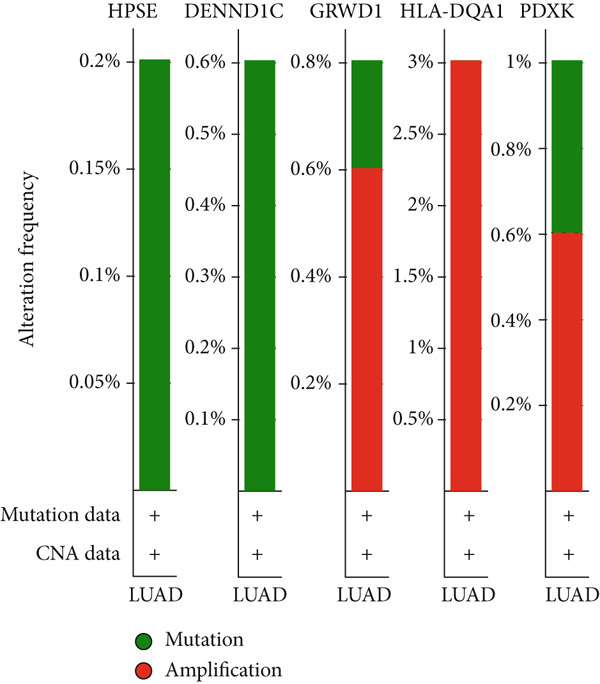
(b)

**Figure:**
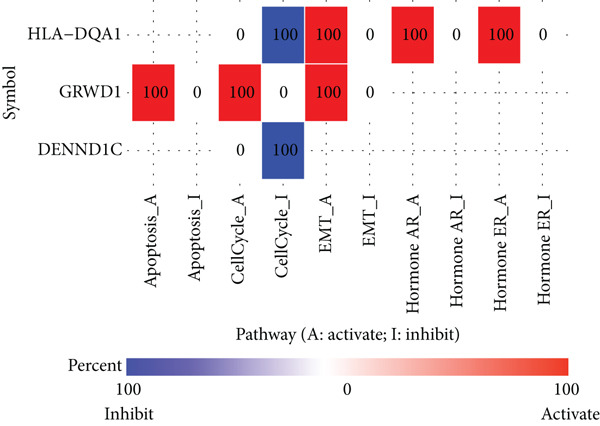
(c)

## 4. Discussion

Platelets have been proposed as major modulators in tumorigenesis, progression, and prognosis of a variety of malignancies, particularly lung cancer [23, 24]. However, there are only several studies to explore the prognostic value of platelet‐derived genes. In this study, we explored lung cancer progression–associated genes from platelet‐derived genes and further developed a platelet‐derived gene signature to predict prognosis and possible response to therapy for patients with lung cancer. Findings from this study provided unique insights into the actions of platelet‐derived genes in prognostic outcomes of lung cancer patients.

WGCNA was employed to identify the genes associated with lung cancer progression [25], which is an algorithm to excavate highly correlated gene modules and to further link these modules with specific clinical traits [26]. In this study, the clinical traits were normal, early, and advanced, and the correlations from “turquoise” module to these three traits were gradually decreased from 0.28 to −0.033. Therefore, genes in “turquoise” module were regarded as being associated with lung cancer progression. The screened disease progression–associated DEGs were primarily involved in ribosome‐related functions, such as rRNA processing, rRNA metabolic process, and ribosome biogenesis. Ribosome is an important organelle for protein synthesis in the cell, and ribosome biosynthesis is a highly complex and conserved process, mainly occurring in the nucleolus [27]. The nucleolus of malignant tumor cells is larger than that of normal cells, and this change is thought to be related to ribosome biosynthesis in the nucleolus [28]. Ribosome biosynthesis runs through the process of tumor occurrence and development [29]. On the one hand, ribosome biosynthesis is essential for cell proliferation, and therefore, infinitely proliferating tumors rely on high levels of ribosome biosynthesis to maintain efficient protein synthesis. On the other hand, various factors and oncogene products that control tumor cell proliferation also regulate ribosome biosynthesis [30, 31]. Therefore, ribosome biosynthesis has become a new hotspot to seek therapeutic strategies for tumors [32, 33]. For example, silencing ribosome biogenesis factor (RBIS) could inhibit cell cycle progression and trigger apoptosis of LUAD cells by inhibiting ribosomal biogenesis, and such an effect was similar and synergistic with CX‐5461, a drug that targets ribosomal biogenesis [34]. Upregulated ribosome biogenesis regulator 1 homolog (RRS1) was negatively linked to the survival of patients with lung cancer, and its silencing could inhibit the malignant behavior of tumor cells and trigger apoptosis of cisplatin‐resistant A549 cells [35]. The above findings confirmed that the identified genes were involved in lung cancer progression by mediating ribosome biosynthesis.

The prognostic value of these disease progression–associated DEGs was further explored, and five crucial genes, including HPSE, DENND1C, GRWD1, HLA‐DQA1, and PDXK, were determined. HPSE, also termed heparanase, is involved in modulating homeostasis of extracellular matrix [36]. Heparanase is highly expressed in all types of lung cancer, particularly LUAD, and its expression showed strong linkage with TNM staging in NSCLC [37] and was negatively correlated with survival of patients with lung cancer [38]. In A549 cells, heparanase exhibited opposite actions with IGFBP3 [39], a potential protective factor in lung cancer [40]. Consistently, expression of HPSE was enhanced in tumor samples in both TCGA and GSE18842 cohorts as well as in the platelet of advanced lung cancer patients. DENND1C encodes connecdenn 3 protein that acts as guanine–nucleotide exchange factors for Rab35, which has been reported to mediate actin polymerization/dynamics and endosomal membrane trafficking [41]. The exact actions of DENND1C have not been investigated in human cancers, including lung cancer. In this study, DENND1C was determined as prognostic genes related to beneficial prognosis in LUAD, which is consistent with the study of Ma et al. [42], who established a 13‐gene signature including DENND1C for prognosis prediction in LUAD. GRWD1 encodes a WD‐repeat protein that is involved in multiple biological processes by interacting with diverse proteins and has been recognized as a potential oncogenic gene in several tumors [43, 44]. For example, GRWD1 is demonstrated to inversely modulate the transcriptional activity of P53 through direct interaction or RPL11‐MDM2 pathway, thus involving in oncogenesis [45, 46]. In addition, GRWD1 is proposed as a crucial ribosome biosynthesis‐related protein involved in tumorigenesis [47]. In lung cancer, enhanced GRWD1 expression is observed in NSCLC tissues, and its high expression is strongly linked to a large tumor size and lymph node metastasis, whereas it is inversely linked to tumor differentiation and survival outcomes [48]. In terms of mechanism, GRWD1 is demonstrated to accelerate NSCLC malignant progression via the activation of the Notch pathway [48]. HLA‐DQA1 belongs to the alpha chain of the human major histocompatibility complex class II (MHC‐II), and the HLA‐DQA1 genotype is closely linked to susceptibility to both LUAD [49] and squamous cell carcinoma [50]. PDXK encodes pyridoxine kinase that phosphorylates vitamin B6 to convert it into pyridoxal‐5‐phosphate, a key cofactor in intermediary metabolism. PDXK is involved in carcinogenesis, progression, and responses to therapy in cancers due to its role in vitamin B6 metabolism [51]. Low PDXK is linked to a worse survival outcome in NSCLC. PDXK is proposed as a biomarker for NSCLC [52]. The Expression of PDXK is positively associated with the infiltration of dendritic cell lysosomal‐associated membrane glycoprotein in NSCLC, thus influencing local immunosurveillance in NSCLC [53].

Based on these five prognostic genes, we established a risk model, which exhibited moderate power for risk stratification in lung cancer patients and was helpful for developing relative individualized treatment. Specifically, the obtained risk score for individuals exhibited close linkages with immune status, with a high‐risk score linked to a low infiltration level of most immune cells and high tumor purity in TME. These results implied that those with a high‐risk score were more likely to have an immunosuppressive TME and might not be candidates for immunotherapy. In addition, the obtained risk score for individuals also correlated with sensitivity to common chemotherapeutics. High‐risk patients had low IC50 to docetaxel [54], gefitinib [55], erlotinib [56], and lapatinib [57], indicating they might benefit from these drugs. The above findings indicated the performance of the five identified genes as biomarkers in lung cancer.

Nevertheless, several limitations should be admitted. Although the signature genes were platelet‐derived, their prognostic value was investigated based on tumor tissue data due to the lack of detailed clinical survival data of samples for platelet RNA‐seq. Differences between tumor tissue and platelet‐derived data may limit the clinical application of the model. Therefore, the performance of this platelet‐derived gene signature should be further investigated based on platelet‐RNA data and corresponding survival data. Besides, all findings from this study were obtained by computational analysis; the expression and role of signature genes in lung cancer progression were not investigated using functional experiments. In subsequent studies, the expression of the identified signature genes should be confirmed in platelet and tumor tissue based on a large scale of clinical samples. The exact roles of these genes in the progression of lung cancer should be investigated by a series of functional experiments both in vitro and in vivo.

## 5. Conclusion

In summary, we identified a set of platelet‐derived genes that were persistently dysregulated along with lung cancer progression, and these genes were primarily involved in ribosome biogenesis–related functional pathways. Five platelet‐derived genes, HPSE, DENND1C, GRWD1, HLA‐DQA1, and PDXK, were identified as key prognostic markers, and the signature developed by these five genes could forecast the survival outcome and possible responses to immunotherapy or chemotherapy. These findings contribute to the development of personalized treatments and the improvement of patient outcomes in lung cancer.

## Ethics Statement

The Ethics Committee of The Second Affiliated Hospital of Xiamen Medical College permitted the application of the human specimens in this research (Approval No. 2018013).

## Consent

The authors have nothing to report.

## Conflicts of Interest

The authors declare no conflicts of interest.

## Author Contributions

L.L.: conception and design of the research, analysis and interpretation of data, drafting the manuscript; X.W.: conception and design of the research, analysis and interpretation of data, drafting the manuscript; K.D.: acquisition of data, analysis and interpretation of data, statistical analysis; M.C.: acquisition of data, statistical analysis; J.X.: acquisition of data, statistical analysis; D.H.: analysis and interpretation of data; Y.Y.: acquisition of data; X.Y.: statistical analysis, obtaining funding; C.‐J.C.: statistical analysis; G.Y.: conception and design of the research, revision of the manuscript for important intellectual content; M.K.: conception and design of the research, obtaining funding, revision of the manuscript for important intellectual content. The manuscript has been approved by all authors for publication, and all authors agree to be accountable for the content and conclusions of the article. L.L. and X.W. are co‐first authors for this study.

## Funding

The study was funded by the Xiamen Science and Technology Plan Project—Lung Cancer Early Detection Technology Based on Platelet RNA Sequencing (3502Z20184060), the Xiamen Medical College Transverse Project (HX202406), and the Xiamen Industry‐University‐Research Collaboration Project (2024CXY0803).

## Data Availability

The data that support the findings of this study are available from the corresponding authors upon reasonable request.
